# Prevalence of hookah smoking and associated factors among male high school students in Iraq

**DOI:** 10.1186/s12889-021-11386-4

**Published:** 2021-07-05

**Authors:** Ahmed K. Al-Delaimy, Waleed A. T. Al-Ani

**Affiliations:** 1 Family & Community Medicine Department / Anbar Medical College, University of Anbar, Ramadi, Iraq; 2 Family & Community Medicine Department Al-Mustansiriya Medical College, University of Al-Mustansiriya, Baghdad, Iraq

**Keywords:** Prevalence, Hookah use, High school students, Risk factors, Iraq

## Abstract

**Background:**

The use of the hookah-smoking device is increasing at a large scale in the Eastern Mediterranean region. Hookah users are exposed to an array of chemical compounds and may suffer several chronic diseases as a result. The purpose of this study was to determine the prevalence of hookah use among male high school students in the region and to study different associated factors in order to provide local tobacco control officials with an understanding of this public health problem.

**Methods:**

A convenient non-probability sampling study was conducted among students in three high schools in Al-Karkh district, Baghdad. The study period was from October 2017 till January 2019 and included 847 male students aged 15–18 years old. Using a simple random technique to select the high schools from a list of schools we chose one school from each directorate. Descriptive, chi-square test of significance, bivariate, and multivariate logistic regression analyses of data were carried out for identifying the risk factors associated with hookah smoking among these high school adolescent males.

**Results:**

The overall prevalence of hookah smoking in the last 30 days among male high school students was 46.1% while in the past 6 months it was as high as 85.7%. More than two-thirds (70.6%) of the students thought that hookah smoking was more socially acceptable than cigarette smoking. Factors such as having first heard about it from friends, the media, or the presence of a hookah café near their residence were significantly associated (*p* > 0.05) with hookah smoking among the students. Similarly, being surrounded by friends who used hookah was also found to be significantly associated with hookah smoking, with an odds ratio of 0.18, 95% CI (0.087–0.394). Hookah smokers were less likely than non-hookah smokers to report its use as forbidden in Islam and more likely to say it is allowed in Islam.

**Conclusions:**

We found an alarmingly high use of hookah smoking among male high school students in this study. Family members and peers had an important role in the prevention of hookah smoking among these students. There is a need for students to be educated about the toxicity of hookah tobacco smoking and its direct effect on their health.

**Supplementary Information:**

The online version contains supplementary material available at 10.1186/s12889-021-11386-4.

## Background

Globally, tobacco use is the second leading cause of mortality, and is responsible for the deaths of 1 in 10 adults [1]. Lung cancer is considered the leading cause of cancer mortality and is linked to the carcinogenic compounds found in tobacco smoke [2, 3]. Nicotine is known to decrease the body’s immune response against malignant growth [4]. In addition to being linked to cancer, tobacco smoke causes several respiratory infections, and is also a factor in hypertension and diabetes, which, among others, are major risk factors for cardiovascular diseases. The effects of each of these risk factors potentiate the risk of cardiovascular events [5–7].

Hookah, also known as Shisha, Hubble-Bubble, Nargileh, and Water-Pipe (WP) smoking, is a common form of tobacco use in the Eastern Mediterranean. Although hookah smoking practices date back at least 400 years, the current use of these devices is increasing at large scale within the region, and is now also increasingly being used in Western countries [8, 9]. Hookah design features a water bowl, hose, and mouthpiece [10]. Several studies have shown that hookah smoking contains harmful chemicals [11–14], and a single 45-min hookah session can expose the smoker to up to 48.6 times the amount of smoke from a cigarette [10, 15]. In the United States (US), a significant increase in the use of hookah among high school students (4.1–7.2%) was reported between 2011 and 2015 [16, 17]. Another study in the US showed that the percentage of both ever and current use of hookah was higher among males than females [18].

Young people have a misconception that hookah smoking is less harmful than cigarette smoking due to the filtration effects provided by the water [19]. However, levels of toxicants in a single hookah tobacco smoke puff can be multiple times higher than found in smoking a single cigarette [10]. These toxic substances include polycyclic aromatic hydrocarbons, nicotine, carbon monoxide, nitric oxides, formaldehyde, and others [11, 13, 14].

Tumbak, Jurak, and Mu’assel are the most widely available types of hookah tobacco, each containing different added substances [20]. Tumbak is unflavored tobacco leaves (Ajami). Jurak contains tobacco, 20% dried fruit, and sugarcane. Both are used widely in Asia and the Middle East. Mu’assel contains 70% sugarcane, glycerol, flavorings, and 30% tobacco [20–22]. The pleasant smell and mild taste from these flavored hookah tobaccos encourages their use, especially among adolescents. Some popular tobacco flavors include fruit, chocolate, spice, alcohol, menthol, candy, sweet, molasses, as well as many others [23–25].

In the Middle East several significant factors have been found to contribute to hookah smoking, including religion, friends and family, low-self-confidence, ready access to hookah devices, and their social acceptability [26–29].

However, there are few studies on hookah smoking behavior in Iraq, and these have mostly been focused on college students [30]. Therefore, our aim was to study the prevalence of hookah use among male high school students in Iraq and the different associated factors linked with hookah smoking with the goal of understanding how health policymakers and the government can approach this growing public health problem.

## Methods

### Study design

A cross-sectional survey in three high schools at Al-Kharkh district west of Baghdad was conducted between October 2018 and the end of December 2018, an area with a population of approximately 100,000 residents.

### Participants

Male high school students aged 15 to 18 years old were eligible to participate in the study, which involved three different year levels, including 4th, 5th, and 6th (equivalent to 10th, 11th, and 12th grade high school levels in the US). A total of 1375 students were therefore invited from the three schools. However, the 6th graders (*n* = 498) were subsequently excluded due to concerns from their school administration regarding participation in the study being potentially disruptive to their important final graduation year. The study participation rate was ultimately 96.5% with 847/877 students completing the questionnaire after 30 students elected to drop out of the study.

### Sampling method

The study used a convenience (non-probability) sample within one of the two Baghdad districts, Al-Kharkh and Risafa. Al-Karkh district, which is similar to Risafa on the opposite side of the Tigris River, was subsequently selected for the study. Although Al-Kharkh has multiple schools in its three educational directorates, due to limitations in available resources for this study, only one school from each directorate could be included. We therefore used a simple random technique to select which of the high schools from a list of schools to include from each. Each directorate has schools at its periphery that are 3–4 km away from its center with the other schools closer together and approximately 1–2 km from the district center. Due to additional security-related factors, we selected schools from each of the three directorates that were closer together, with 8 such schools within one directorate, 10 such schools in the second directorate, and 10 such schools in the third directorate.

### Study size

The study size was based on the sampling equation: (*n* = Z^2^ 1-α/2 P(1-P)/d^2^) where *n* is the required sample size, Z^2^ 1-α/2 is the confidence interval (95% = 1.96, *P* = 0.44 estimated proportion), d is the 0.025 desired precision [31, 32]. This resulted in a sample size of 847 students included in the study, with a level of significance at 5%.

### Questionnaire

Before circulating the final version of the questionnaire to the students, a pilot assessment was carried out among 28 students in one of the three schools (Al-Quds High School) to check the suitability of the Arabic questionnaire version.

The questionnaire was based on the California Tobacco Surveys for tobacco use [33] and modified to the social context of Iraq. It was translated from English to Arabic at the College of Literature in the Department of English at the University of Anbar. A questionnaire form was prepared with areas of questioning including, for example, the number of times that hookah tobacco was smoked in the past 30 days, how many hookah smoking sessions/day occurred among regular users, the level of confidence that the person felt they could quit hookah smoking, when the first time they heard about hookah was, their opinion about hookah smoking and Islam, and if they support regulations to close hookah cafés. The self-administered questionnaire was distributed to students by the first author, AKAD, to be completed in pencil and collected during class time. The survey was anonymous to protect privacy and ensure confidentiality. Questionnaire (English version) was added as supplementary file.

### Statistical methods

Analysis of data was carried out using SPSS-25 (Statistical Packages for Social Sciences- version 25). The significance of the difference between percentage results (descriptive data) was tested using the Pearson chi-square test (χ^2^-test) with the application of Yate’s correction or Fisher exact test as applicable. Bivariate application and multivariate logistic regression analysis of data were carried out for comparison between independent variables among those who smoke hookah. All significant variables (*p* < 0.05) in the bivariate analysis were included in the multivariate analysis. Multivariate logistic regression analysis was used to investigate the significant risk factors for hookah smoking. The significance level for all the analyses was set at *p* < 0.05. In logistic regression, the variable of “smoked hookah tobacco in the past 30 days” was the dependent variable and the categorical covariates (with a reference category for each covariate) were “first heard about hookah”, “there are people surrounding students that smoke hookah”, “the types of smoking that are most harmful to health”, “is hookah smoking acceptable socially more than cigarette smoking”, “hookah smoking is less harmful and less addictive than cigarette smoking”, “student’s opinion about hookah smoking in Islam”, and “support regulations to close hookah cafés”**.**

### Ethical issues

Ethical clearance was obtained from both the ethical committee at Anbar University Medical College and the Directorate of Education at Al-Karkh district.

The ethics committee waived the need for written informed consent from the parents of study participants. Permission was obtained from the three school administrations to do the survey. School administration informed students verbally before the survey started that they would be asked to complete a questionnaire survey related only to hookah in the coming days, and that students would need to inform their parents. Direct verbal assent was also sought from the parents of high school students below the age of 16. Written informed consent was obtained from the high school students, and they had the right to participate or refuse/withdraw from the study.

## Results

The study participants consisted of 847 male high school students. The response rate was 96.5%. Almost all data were normally distributed except for the variable ‘regulation to close hookah café places’ which had positive skewness (longer tail on the right).

### The prevalence of hookah smoking among students

The prevalence of those who smoked hookah tobacco in the past 30 days was (46.1%) (95% CI = 42.8–49.9). We also found that 57 students had tried hookah smoking by having at least one puff (6.73%) 95% CI (4.9–8.5).

Hookah smoking among students in the past 6 months was 85.7%, while more than a quarter (28.1%) had tried hookah once in the last month (see Table 1). The highest percentage of students smoking hookah was among the ages of 15 and 16 (26.3% & 27.4%, respectively). While the highest duration of smoking during each session was 30–44 min and 60–119 min, 24.3 and 35.6% respectively (see Table 1).

**Table 1:** Descriptive characteristics associated with hookah smoking (last 6 months), duration of hookah sessions, and other variables

Variable & Category	Frequency smoked	n	%
Number of times smoked hookah tobacco in the past 30 days.	1	110	28.1
	2	65	16.6
	3	44	11.3
	4	16	4.1
	5	23	5.9
	6	6	1.5
	7---13	48	12.4
	14---20	32	8.3
	21---27	3	0.9
	28---49	32	8.2
	≥50	12	3.4
Hookah smoked in the last 6 months	Yes	335	85.7
	No	56	14.3
Age first smoked hookah	< 10 years	6	1.5
	10	14	3.6
	11	7	1.8
	12	19	4.9
	13	30	7.7
	14	59	15.1
	15	103	26.3
	16	107	27.4
	≥17 years	46	11.8
Hookah smoking sessions/day among regular users	One	52	13.3
	Two	134	34.3
	Three	121	31.0
	Four	81	20.7
	Five & more	3	0.8
Sharing mouth-piece of hookah-smoking device	Never	120	30.7
	Sometimes	135	34.6
	Most of the times	59	15.1
	Always	77	19.7
Student’s confidence that he can quit hookah smoking?	Completely confident	239	61.2
	Confident	83	21.3
	Some Confident	30	7.7
	Not that Confident	22	5.6
	Not Confident at all	17	4.3
Duration of hookah smoking at each session (minutes)	< 15 min	58	15.0
	15–29	20	5.1
	30–44	95	24.3
	45–59	24	6.2
	60–119	139	35.6
	≥120 min	55	14.2

### Social and cultural factors associated with hookah use

Table 2 presents results on social and cultural factors associated with hookah use. The highest percentage of students first learned about hookah from friends (55%) and most (86.1%) knew that there was a café shop for hookah smoking near their residence. We also found that friends were the most common group reported to smoke hookah near students (65.2%) (see Table 2).

**Table 2:** Social and cultural factors associated with hookah users, including Islam’s opinion about hookah use, social acceptance, and other variables

Variable		n	%
First, heard/know about Hookah?			
Father & Mother		36	4.3
Brothers, Sister & Cousin		143	16.9
Friends		466	55.0
Media and Newspaper		73	8.6
Saw a Hookah Café shop		120	14.2
Others (internet)		40	4.7
Presence of Cafe for hookah smoking around students’ residence		729	86.1
People surrounding/near students that smoke hookah.			
Father &Mother		38	4.5
Brothers &Sisters		69	8.1
Other close relatives		268	31.6
Friends		552	65.2
None		76	9.0
Which of the following types of smoking are more harmful to health?	Tobacco gum	50	5.9
	Cigarette	293	34.6
	e-cigarette	202	23.8
	Hookah	302	35.7
Student’s opinion for Hookah smoking in Islam.	Forbidden	177	20.9
	Discouraged	401	47.3
	Allowed	79	9.3
	Do not know	190	22.4
Is hookah smoking acceptable socially more than Cigarette smoking?	Yes	598	70.6
	No	249	29.4
Is hookah smoking is less harmful and addictive than cigarette smoking?	Yes	314	37.1
	No	533	62.9
Agree on regulations to forbid café places?	Yes	690	81.5
	No	157	18.5

### Islam’s opinion regarding hookah smoking, socially acceptance and agreement on the regulation

In terms of the Islam’s opinion regarding hookah smoking, students mostly reported this being either discouraged (47.3%) or forbidden (20.9%) (see Table 3).

**Table 3:** Bivariate analysis for association of different factors affecting hookah smoking

		Hookah smoking (even one puff)		*P*-value	OR (95%CI)
		Yes (*n* = 391) %			
From whom student first heard/know about Hookah?					
Father &Mother		4.6		0.637	1.17 (0.60–2.29)
Brother, Sister & Cousin		18.4		0.271	1.22 (0.85–1.75)
Friends		59.6		0.013*	1.41 (1.07–1.85)
Media and Newspaper		5.9		0.009*	0.51 (0.30–0.85)
Saw a Hookah Café shop		13.3		0.502	0.88 (0.59–1.29)
Web Networks		3.6		0.147	0.61 (0.32–1.19)
Presence of Cafe for hookah smoking around students’ residence		90.5		0.0001*	2.07 (1.36–3.13)
People surrounding/near students that smoke hookah.					
Father & Mother		5.9		0.069	1.84 (0.95–3.57)
Brother & Sister		10.2		0.040*	1.68 (1.02–2.76)
Other close relatives		33.5		0.280	1.17 (0.88–1.57)
Friends		72.1		0.0001*	1.78 (1.33–2.38)
None		3.3		0.0001*	0.22 (0.12–0.40)
Which of the following types of smoking are more harmful to health?	Tobacco gum	6.6		0.0001*	0.78 (0.43–1.43)
	Cigarette	43.5			Ref
	e-cigarette	24.3			0.64 (0.45–0.92)
	Hookah	25.6			0.36 (0.26–0.50)
Student’s opinion for Hookah smoking in Islam.	Forbidden	13.6		0.0001*	0.44 (0.28–0.67)
	Discouraged	48.6			0.92 (0.65–1.30)
	Allowed	13.8			2.21 (1.27–3.84)
	Do not know	24.0			Ref
Is hookah smoking acceptable socially more than Cigarette smoking?		0.8		0.0001*	2.60 (1.90–3.56)
Think that hookah smoking is less harmful and less addictive than cigarette smoking?		55.0		0.0001*	4.41 (3.27–5.94)
Agree on regulations to forbid café places		72.4		0.0001*	0.32 (0.22–0.46)

*Significant association using Pearson Chi-square test at 0.05 level

More than two-thirds (70.6%) of students believe that hookah smoking is more acceptable socially than cigarette smoking. There was agreement among more than three-quarters of students of the need for regulations to close or ban hookah cafés (81.5%) (see Table 2).

### Bivariate analysis on the associations between related factors and hookah smoking

The bivariate analysis demonstrated that the subgroup variables friends, media, and the presence of a hookah café near the student’s residence were associated with students being more likely to smoke hookah compared to other subgroups in the same variable (first heard). Students were most likely to be using hookah if they had heard about it from friends (OR 1.4 (95% CI = 1.07–1.85)), while those who heard about hookah from the media were less likely to use it (OR = 0.5, 95% CI = 0.30–0.85) (see Table 3). Among the different groups of people in a student’s life who smoke hookah we found that friends who smoked (OR 1.78 (95% CI = 1.33–2.38) and a brother or sister who smoked were significant risk factors for students to be a hookah smoker themselves (OR 1.68 (95% CI = 1.02–2.76) respectively)).

Also, we found that students were more likely to smoke hookah if there was a hookah café near their residence (OR = 2.0 (95% CI: 1.36–3.13)). Among the four types of smoking listed on the survey, students believed that cigarettes have a more hazardous effect on human health than hookah (43.5% Vs. 25.6%; OR = 4.4). Those reporting that hookah smoking was accepted socially were much more likely to be hookah smokers (OR = 2.6), while those supporting regulation to close café places were less likely to be hookah smokers (OR = 0.3) (see Table 3).

### Multivariate analysis of the associations between related factors and hookah smoking

Table 4 presents results that reveal the different risk factors associated with hookah smoking. This table includes all variables in the model based on the univariate results from Table 3. Results clearly indicate students who had family and friends who smoked hookah in their presence were more likely to also use it, although only the variable of having friends who smoked hookah reached statistical significance. Having a coffee shop that sells hookah near their house was also significantly associated with hookah use. If the students thought hookah is less harmful or more socially acceptable they were significantly more likely to use hookah (see Table 4).

**Table 4:** Multivariate logistic regression of risk factors affecting hookah smoking

Reference Category was the (first).	*P* value	Odds Ratio	95% C.I. for odds ratio	
			Lower	Upper
**First heard/know about Hookah?**				
Brother, Sister and cousin	.084	2.310	.894	5.964
Friends	.035*	2.635	1.069	6.496
Media & Newspaper	.558	1.348	.496	3.664
Saw a Hookah Café shop	.201	1.835	.724	4.651
Web Networks	.101	2.711	.823	8.931
**People surrounding/near students that smoke hookah.**				
Brother & Sister	.183	1.513	.823	2.782
Close relatives	.173	1.345	.878	2.059
Friends	.017*	1.770	1.110	2.824
None	.027*	.374	.157	.895
**There is a cafe for WP smoking around your residence**	.005*	2.817	1.362	5.827
**Which of the following types of smoking are more harmful to health?**				
Cigarette	.620	.837	.415	1.690
e –cigarette	.991	1.004	.486	2.075
Hookah	.316	1.437	.707	2.920
**Is hookah smoking acceptable socially more than Cigarette smoking??**	.000*	1.996	1.389	2.868
**Hookah smoking is less harmful and less addictive than cigarette smoking?**	.000*	3.703	2.656	5.162
**Student’s opinion for Hookah smoking in Islam.**				
Discouraged	.002*	.512	.335	.782
Allowed	.000*	.314	.165	.598
Don’t Know	.007*	.513	.315	.836
**Agree on regulations to forbid café places.**	.000*	.391	.257	.594

*Significant association using Pearson Chi-square test at 0.05 level

## Discussion

This is the first study to investigate the risk factors associated with hookah smoking among male high school students in Iraq. We found that although most students who smoked hookah had an accurate perception of how Islam views hookah smoking as forbidden or harmful, and most supported regulations against hookah cafés, the overall prevalence of hookah smoking among the students was high (46%). This high rate of hookah use was similar to that found in a study of hookah use in secondary school adolescent males, aged > 18 years, in Saudi Arabia, which was 44% [32]. However, studies reported by Abbas et al and Alzyoud et al in Iranian and Jordanian male high school students found a different, much lower rate of 6 and 24%, respectively [34, 35]. The high rate reported in the current study might reflect the acceptance and spread of such harmful behavior in such a young age group due to recent problems of conflict imposed on Iraq, making tobacco control less of a priority by the government. Our study is also the first to explore the multiple social factors associated with smoking hookah in this age group and also the first to consider the role of religion in this behavior. This can help provide health policymakers with the tools they need to develop policies or interventions to tackle this growing and serious public health problem. Our findings may also be applied to the Middle East as a whole, since the region shares is a similar culture and language and the use of hookah is increasing throughout.

More than half of participants first heard about hookah smoking from friends and more than two-third have friends who smoke hookah, which highlights the role of friends in initiating hookah smoking. Similar findings were reported by Azodi et al in Iran and Bejjani et al in Lebanon [36, 37]. This points to the fact that a new generation of hookah users is present, unlike previous generations where it was not smoked as commonly as now.

Among different types of tobacco smoking, high school students in this study thought that hookah smoking caused more harm to health than nicotine gum or electronic cigarettes. Aslam et al. in their review explained the significant association between hookah smoking and increased risk of heart disease, cancer, and hypercholesterolemia [38]. Although adolescents in our study believe that hookah smoking had worse health effects than other types of smoking, they still used it. We believe this is likely due to the relatively pleasant aroma of the smoke, flavor, and taste of hookah smoking that can overcome an individual’s perception of its harm. We believe this may also be influenced by peer pressure as hookah devices can bring groups of friends or other acquaintances together socially. This could explain the reason hookah smoking is seen as more acceptable socially than cigarette smoking. This agrees with findings from Fitzpatrick et al. in the USA, Momenabadi et al in Iran and Tamim et al in Lebanon who reported attempts of hookah smoking among young adult users were associated with the belief that it is socially and culturally more acceptable than cigarette smoking [27, 39, 40], and this led to an increase in the prevalence of this behavior. Hookah may therefore present a greater challenge to changing social norms around its acceptance in society.

A high percentage of students believed hookah smoking was discouraged and forbidden in Islam (68.2%) reflecting the important role that religion and faith can play in preventing smoking, as it does in regards to other risky behaviors such as gambling, drug abuse, and alcohol drinking [41]. A study from Jordan which looked into smoking habits among university students and academics at different levels found that individuals in the faculty of religious studies were less likely to smoke compared to those in other faculties [42].

Sajid et al. reported that carboxyhemoglobin concentration in cigarette smokers is lower (6.1 ppm) than in individuals who smoke shisha (8.8 ppm) [43]. In the current study we found that more than three-quarters of adolescent hookah smokers reported smoking for 30 min or more at each session. This is concerning since duration of hookah smoking sessions, depth of inhalation, and frequency of puffing all contribute to the level of exposure to nicotine and other carcinogenic chemicals present in the charcoal and tobacco in shisha smoke. As mentioned above, hookah has high concentrations of a carboxyl group, nicotine, and other chemicals, even higher than cigarette smoking, and has an addictive impact on the body causing withdrawal symptoms when trying to quit. Despite this, three-quarters of the students in the current study reported that they can quit hookah smoking when they decide to.

Our multivariate logistic regression model showed a significant difference among subgroups who smoked around the students, reflecting the greater influence of family and peers on adolescent health and behavior [44]. Hookah use was the only significant sub-variable among the others in comparison with the reference category e-cigarette. Also, hookah use was higher among those who said hookah smoking was socially acceptable and less harmful than cigarette smoking.

### Limitations and strength of the study

Only one school from each directorate was selected to participate in this study due to the limitations in financial support and issues with security. Therefore, our use of a convenience sample may have impacted the generalizability of the study, although we believe that our results are representative of students from elsewhere in the district given there are similar social norms across the Iraq [45]. Although the Arabic version of the questionnaire was not validated, we did pretest the questionnaire prior to the main study and necessary modifications were made. In all three high schools, the school administration declined to enroll the sixth-grade (final graduation year) students in the study since they did not want to divert any student attention away from the task of completing their final and most difficult year prior to graduating on to University or College. If we had been able to include the sixth grade students we believe the factors we found associated with hookah use may have become more significant. Since the study was limited to male students, socio-demographic characteristics were not included in the questionnaire. Hookah is rarely smoked by females of high school age. Also, the study may have led to under reporting of use [45] given hookah and cigarette smoking in high school is not accepted legally. However, the students completed their questionnaires anonymously and thus we may assume their responses were candid.

### Policy implications

School-aged children and other youth are the most appropriate group for intervention programs aimed at preventing hookah smoking. We believe this can be facilitated by carrying out regular surveys, for example every 2 years, with representative samples of young people in schools to assess prevalence of hookah smoking in this group and track its associated factors. Education techniques can also be used that are tailored to the socio-culture of even younger children (primary school level). Furthermore, socially based rather than information based prevention programs can be implemented to address the specific needs of particular youth cultures known to promote hookah use. It is also necessary to work with government to introduce a total ban on advertising of hookah smoking and ensure legislation is in place to limit hookah cafés and overcrowding. Overall, a comprehensive national and community strategy is needed to take action on the social, interpersonal and personal influences that lead to hookah use in youth.

## Conclusions

This study reported an alarmingly high use of hookah tobacco smoking among male high school students in Iraq, and that it is an acceptable socio-cultural phenomenon among them. Effective control measures are required including health education, school intervention programs, and legislation against hookah cafés. Further studies are needed to compare similarly aged youth across the region and to understand the differences and similarities of this behavior and its public health impact.

## Supplementary Information

**Additional file 1.** Hookah Questionnaire (English version) was added as supplementary file.

## Data Availability

‘The datasets used and/or analysed during the current study are available from the corresponding author on reasonable request.’

## Abbreviations

KAP: Knowledge, Attitude, Practice
OR: Odds Ratio
C.I: Confident Interval
WP: Water Pipe
e-cigarette: Electronic cigarettes
