# Supplementary material for: Prevalence of hookah smoking and associated factors among male high school students in Iraq
Source: BMC Public Health. 2021 Jul 5;21:1317. doi: 10.1186/s12889-021-11386-4 (PMC8256481; doi:10.1186/s12889-021-11386-4)
Supplement: Supplementary file 1 — Additional file 1. Hookah Questionnaire (English version) was added as supplementary file. [file 12889_2021_11386_MOESM1_ESM.docx]

**Additional files**

**Hookah Questionnaire (English version) was added as supplementary file.**

**Questionnaire form**

Informed consent to participate in this survey:

Signature ________________

Dear student,

Place a circle on the write answer. Don’t’ put your name on the form.

---------------------------------------------------------------------

1. First, heard/know about Hookah?

1 – My parents 2 - Brothers, sisters, cousins 3 - friends 4 - news and newspapers 5-saw hookah Shop 6- Others; ____________

2- Presence of Cafe for hookah smoking around students’ residence?

1. Yes 2. No 3. I do not know

3 - Do you know from those mentioned below who smokes hookah?

1 – Parents 2 - brothers and sisters 3 – close relatives 4 - friends 5 – none

4- Which of the following types of smoking is more harmful to health?

1-Tobacco gum 2-cigeratte 3-electronic cigarettes 4-hookah

5 - Do you think that hookah smoking acceptable socially more than Cigarette smoking?

1. Yes 2. No

6 - In your opinion, do you think hookah smoking is less harmful and addictive than cigarette smoking?

1-Yes 2-No

7 – Do you think Hookah smoking in Islam is:

1 – Forbidden (haram) 2 - Discouraged 3 - allowed 4 - do not know

8 – Do you agree on regulations to forbid café places?

1. Yes 2. No

9 - Have you smoked hookah in the past 30 days (even one puff)?

1-Yes 2-No

If yes, how many times you smoked hookah tobacco in the past 30 days? ------------

10- Have you ever smoked hookah during the last 6 months?

1-Yes 2-No

11- How old are you when you first smoked hookah? _________

12- On average, how many sessions per day you smoke hookah?

1-once 2-twice 3- three time 4- four times 5- five times and more

13. When you have smoked hookah last time, have you shared the mouth-piece of hookah-smoking device?

1-Never 2-Sometimes 3-Most of the times 4-Always

14 - How confident you are that you can quit hookah smoking?

1.Completely confident 2. Confident 3.Some confident 4. Not that confident 5. Not confident at all

15. When you smoke hookah how long does each session take from you?

     ___________________ Enter a number in minutes.

**Questionnaire form (in Arabic)**

**(عزيزي الطالب ضع دائرة على الاختيار المناسب) *****( لا تكتب اسمك على الاستبيان)**

**ملاحظة: للطالب حق الانسحاب من البحث او عدم اكمال الاستمارة**

**توقيع الطالب بموافقته باجراء الاستبيان__________**

**--------------------------------------------------------------------------------**

**1-من اين سمعت وعلمت عن النركيلة (الشيشة) اول مرة؟**

**1-من الوالدين 2-من اخوتي والاقرباء 3-من اصدقاء 4-من الاخبار والصحف**

**5-رايت مقهى للنركيلة 6-اخرى؛ حدد___________**

**2- هل يوجد مقهى للنركيلة في منطقتكم ؟**

**1-نعم 2-لا 3- لااعلم**

**3- هل تعلم من المذكورين ادناه من يدخن نركيلة؟**

**1-الوالدين 2-الاخوة والخوات 3-اقرباء اخريين 4-اصدقاء 5-لايوجد احد**

**4- في رايك اي من المذكور ادناه هو الاكثر ضررا على الصحة:**

**1-علك تبغ 2-سكائر 3-السكائر الالكترونية 4-نركيلة**

**5- هل تعتقد ان النركيلة اجتماعيا مقبولة اكثر من السكائر؟**

**1-نعم 2- كلا**

**6- هل تعتقد ان النركيلة اكثر امانا واقل ادمانا وضررا من السكائر؟**

**1-نعم 2-كلا**

**7- في رايك هل تدخين النركيلة في الاسلام:**

**1-حرام 2- مكروه 3- مسموح (مباح) 4- لاادري**

**8-هل تؤيد وضع قوانين تمنع مقاهي النركيلة؟**

**1- نعم 2- كلا**

**9-هل سبق ان دخنت النركيلة خلال ال30 يوم الاخيرة ولو شفطة واحدة ؟**

**1-نعم 2-كلا**

**10- هل سبق ان دخنت النركيلة او الشيشة خلال الستة اشهر الماضية؟**

**1-نعم 2-كلا**

**11- كم كان عمرك عندما دخنت نركيلة اول مرة؟ _________**

**12- كمعدل كم مرة تدخن النركيلة باليوم؟**

**1 مرة واحدة 2-مرتين 3- ثلاث مرات 4-لربع مرات 5- خمس مرات واكثر**

**13- هل تشارك نفس قطعة فم النركيلة مع الاخرين؟**

**1-ابدا 2-احيانا 3-اغلب الاوقات 4-دائما**

**14- كم هي ثقتك بنفسك انك تستطيع ايقاف تدخين النركيلة؟**

**1-كلي ثقة 2-توجد ثقة 3-توجد بعض الثقة 4-ليس جدا واثق**

**5-ليس واثق ابدا**

**15- عندما تدخن النركيلة كم تستغرق وقت الجلسة؟**

**___________________ اكتبها رقما بالدقائق**
